# Rapid activation of epithelial-mesenchymal transition drives PARP inhibitor resistance in *Brca2*-mutant mammary tumours

**DOI:** 10.18632/oncotarget.26830

**Published:** 2019-04-05

**Authors:** Liliana D. Ordonez, Trevor Hay, Robert McEwen, Urszula M. Polanska, Adina Hughes, Oona Delpuech, Elaine Cadogan, Steve Powell, Jonathan Dry, Giusy Tornillo, Lucy Silcock, Elisabetta Leo, Mark J. O’Connor, Alan R. Clarke, Matthew J. Smalley

**Affiliations:** ^1^ European Cancer Stem Cell Research Institute, Cardiff University, Cardiff, UK; ^2^ Oncology, IMED Biotech Unit, AstraZeneca, Cambridge, UK; ^3^ Oncology, IMED Biotech Unit, AstraZeneca, Waltham, MA, USA; ^*^ Posthumous authorship

**Keywords:** PARP inhibitors, olaparib, Brca2, EMT, P-gps

## Abstract

Tumours defective in the DNA homologous recombination repair pathway can be effectively treated with poly (ADP-ribose) polymerase (PARP) inhibitors; these have proven effective in clinical trials in patients with *BRCA* gene function-defective cancers. However, resistance observed in both pre-clinical and clinical studies is likely to impact on this treatment strategy. Over-expression of phosphoglycoprotein (P-gp) has been previously suggested as a mechanism of resistance to the PARP inhibitor olaparib in mouse models of *Brca1/2*-mutant breast cancer. Here, we report that in a *Brca2* model treated with olaparib, P-gp upregulation is observed but is not sufficient to confer resistance. Furthermore, resistant/relapsed tumours do not show substantial changes in PK/PD of olaparib, do not downregulate PARP1 or re-establish double stranded DNA break repair by homologous recombination, all previously suggested as mechanisms of resistance. However, resistance is strongly associated with epithelial-mesenchymal transition (EMT) and treatment-naïve tumours given a single dose of olaparib upregulate EMT markers within one hour. Therefore, in this model, olaparib resistance is likely a product of an as-yet unidentified mechanism associated with rapid transition to the mesenchymal phenotype.

## INTRODUCTION

Cancer cells with specific defective DNA-damage response pathways show synthetic lethality with inhibition of PARP, a key enzyme in single strand break repair [1, 2]. PARP inhibitors, such as olaparib, harness this principle to selectively kill BRCA-deficient cells. Olaparib has demonstrated excellent anti-tumour activity in *Brca*-mutated breast cancer models [3, 4] and clinical trials in *BRCA*-mutated cancer patients have also proven efficacious, leading to the approval of olaparib in over 60 countries world-wide (https://www.lynparza.com). Both preclinical and clinical evidence also suggest activity in non-BRCA homologous recombination repair (HRR) defective backgrounds [5, 6].

The synthetic lethal interactions of olaparib with BRCA1 and BRCA2 defects remain a key area of interest. Resistance to olaparib has been seen in pre-clinical *Brca*-mutant mammary models [3, 4] and has also been reported in the clinical setting [7]. Elucidation of the diverse mechanisms of resistance to PARP inhibition is imperative, so that new approaches for more accurate patient stratification for potential novel combinations or follow-up therapies may be identified.

Here, we have investigated the effects of olaparib in an established *BlgCre Brca2/Tp53-*mutant mouse mammary model [3]. We analyzed a number of established olaparib-resistance mechanisms, including up-regulation of efflux pumps [4, 8], restoration of HRR [7, 9, 10] and loss of PARP1 [11]. We have found that the initial response to olaparib treatment is a very rapid (within one hour of treatment) activation of a mesenchymal-like differentiation program and, indeed, that either an initial weak response to olaparib treatment, or acquired resistance following an initial good response, correlated primarily with epithelial-to-mesenchymal transition (EMT), where epithelial cells lose their cell-cell adhesion and apical-basal polarity and convert into mesenchymal-like cells. Tumours with EMT features have been shown to be highly resistant to chemotherapies [12, 13] and our data suggest that EMT is associated with both intrinsic and acquired resistance to olaparib. As previously reported, up-regulation of P-gp was also associated with resistance [3, 8]. However, inhibition of efflux pumps in our olaparib-resistant tumours had no effect on relapse and resistant/relapsed tumours did not have substantial changes in olaparib PK/PD or re-establish HRR [10]. Therefore, in this model, olaparib resistance is likely a product of an as-yet unidentified mechanism associated with the mesenchymal phenotype.

## RESULTS

### Treatment-naïve *BlgCre Brca2/Tp53-*mutant tumours show a range of histological phenotypes

**Figure 1:**
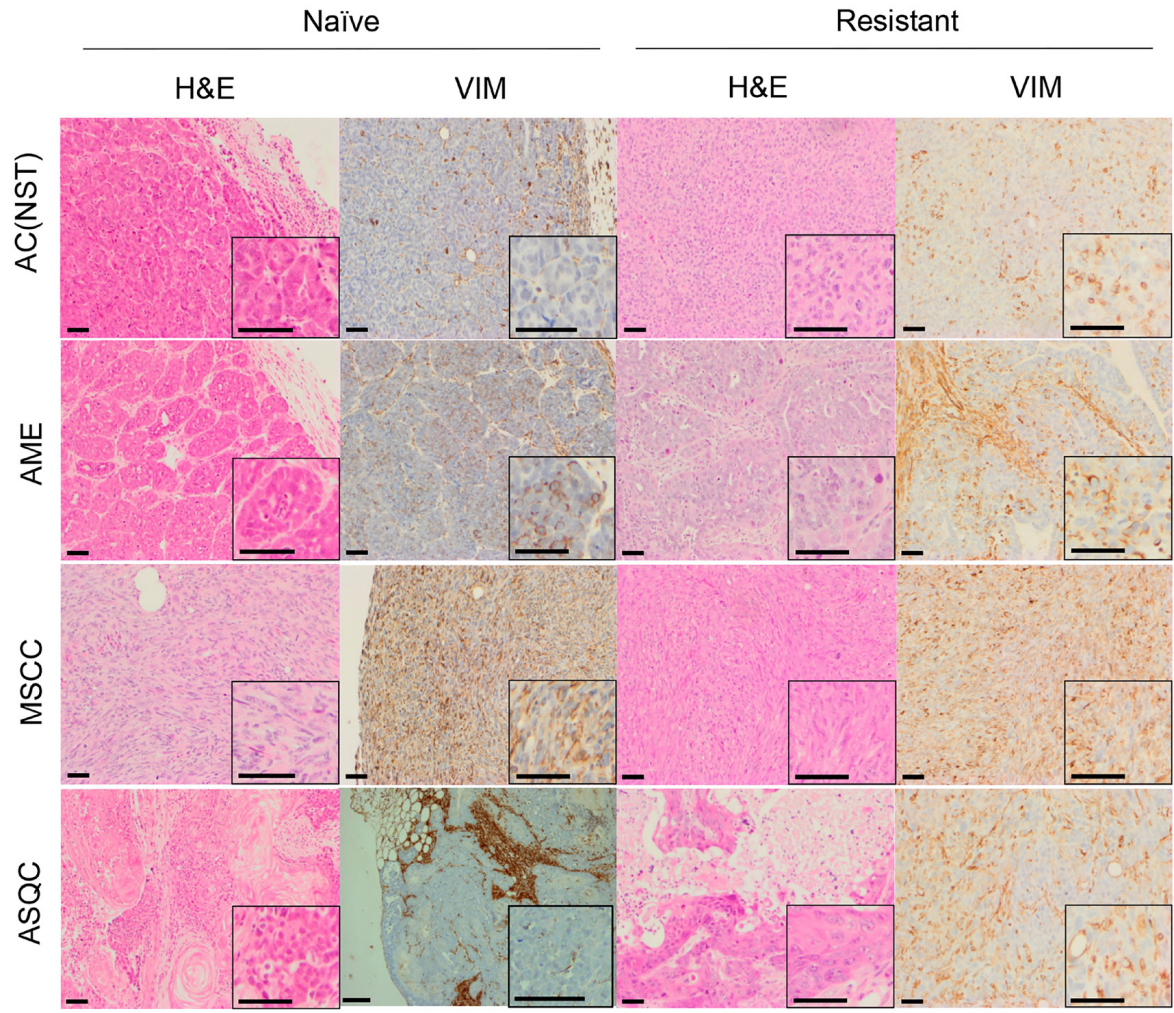
H&E and vimentin staining of tumour phenotypes observed in olaparib-naïve and olaparib-resistant tumours in the *Brca2/p53*-mutant mammary tumour model Representative pictures of tumour sections stained by H&E, or with an antibody against VIM. Scale bars = 50µm. AC(NST) = adenocarcinoma (No Special Type); AME = adenomyoepitheliomas; MSCC = metaplastic spindle cell carcinoma; ASQC = adenosquamous carcinoma.

**Figure 2:**
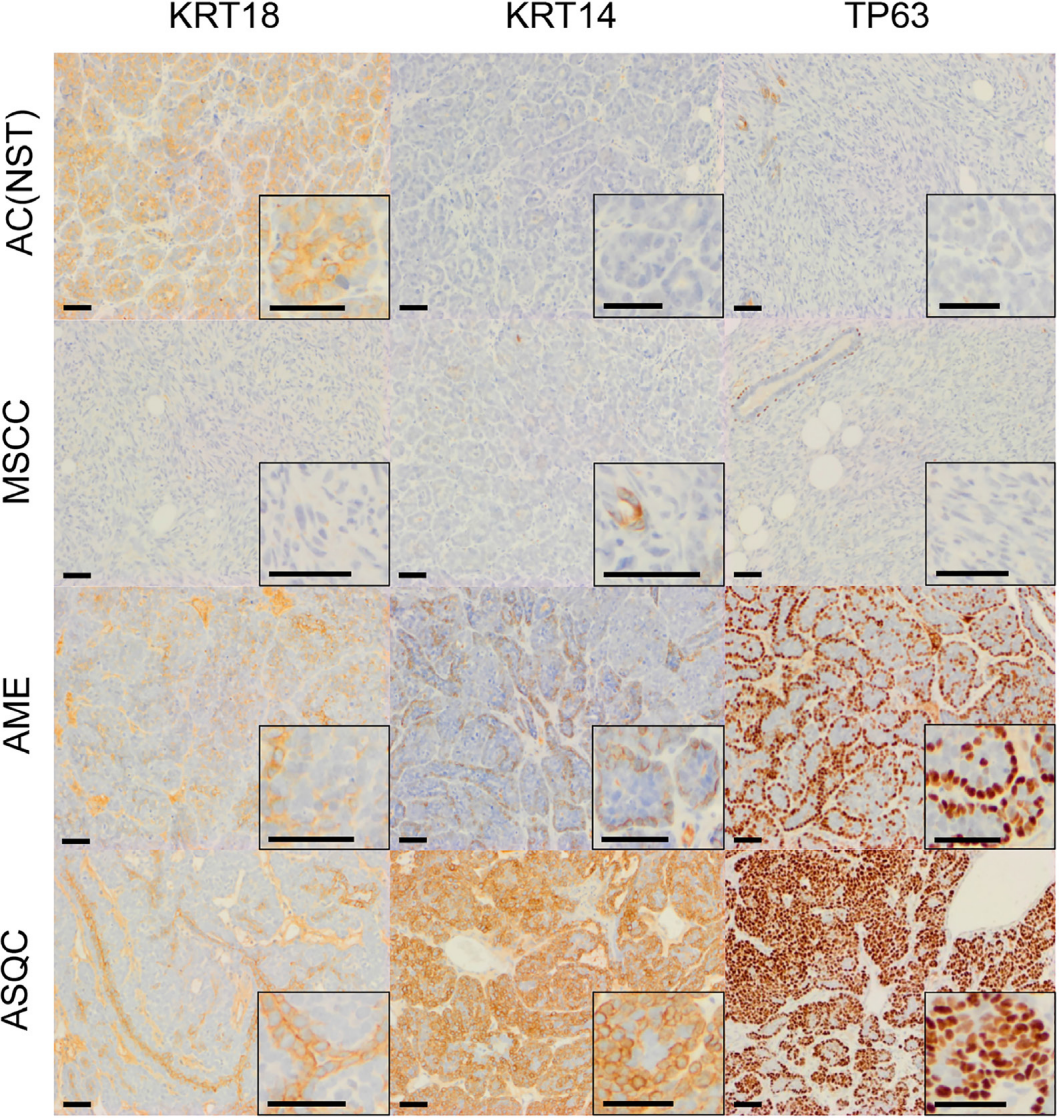
Immunohistochemical staining of olaparib-naïve tumours of different phenotypes in the *Blg-Cre Brca2/p53*-mutant mammary tumour model Representative pictures of tumour sections stained with antibodies against either KRT18, KRT14 or TP63. Scale bars = 50 µm.

**Figure 3:**
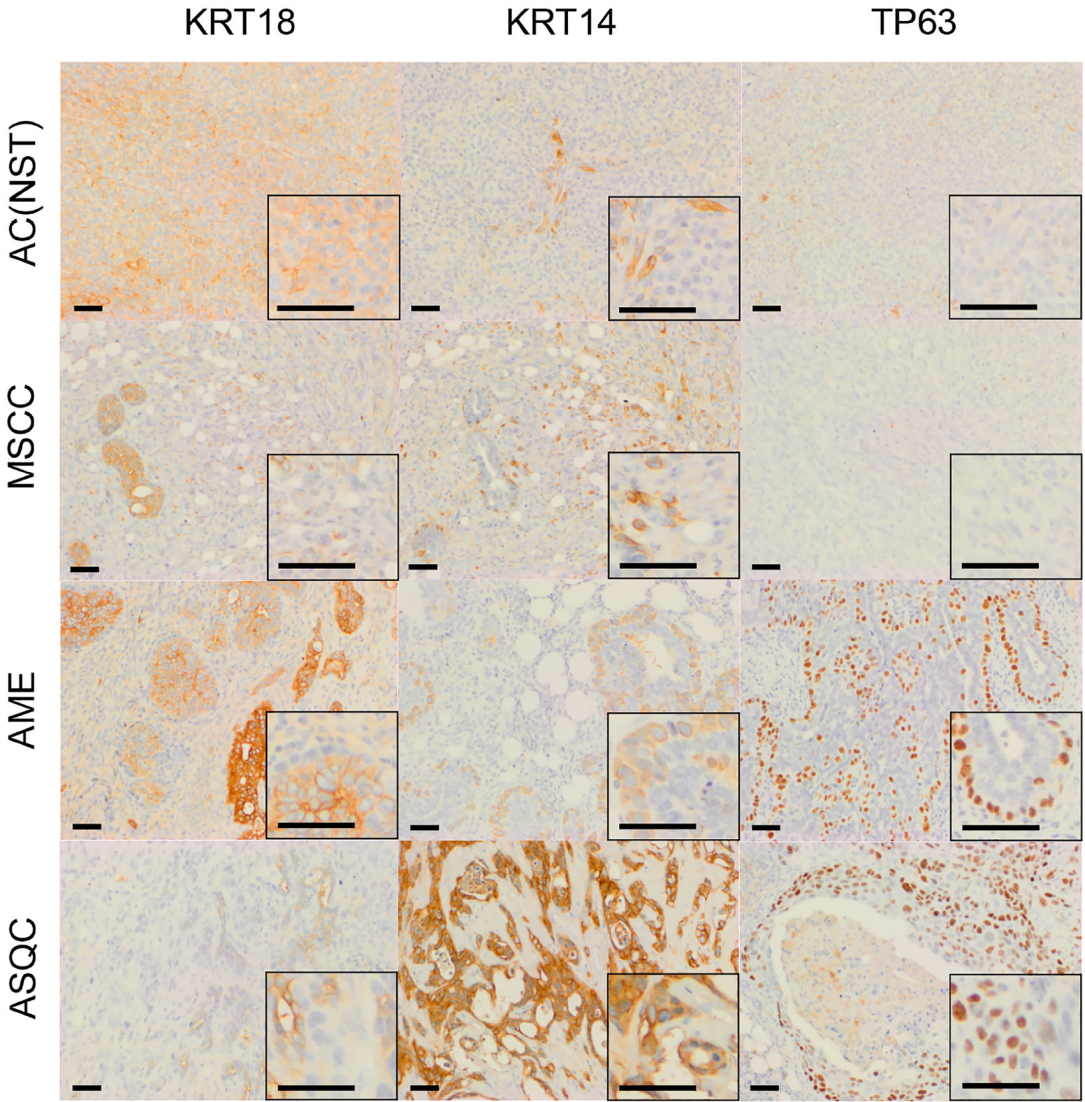
Immunohistochemical staining of olaparib-resistant tumours of different phenotypes in the *Brca2/p53*-mutant mammary tumour model Representative pictures of tumour sections stained with antibodies against either KRT18, KRT14 or TP63. Scale bars = 50 µm.

**Figure 4:**
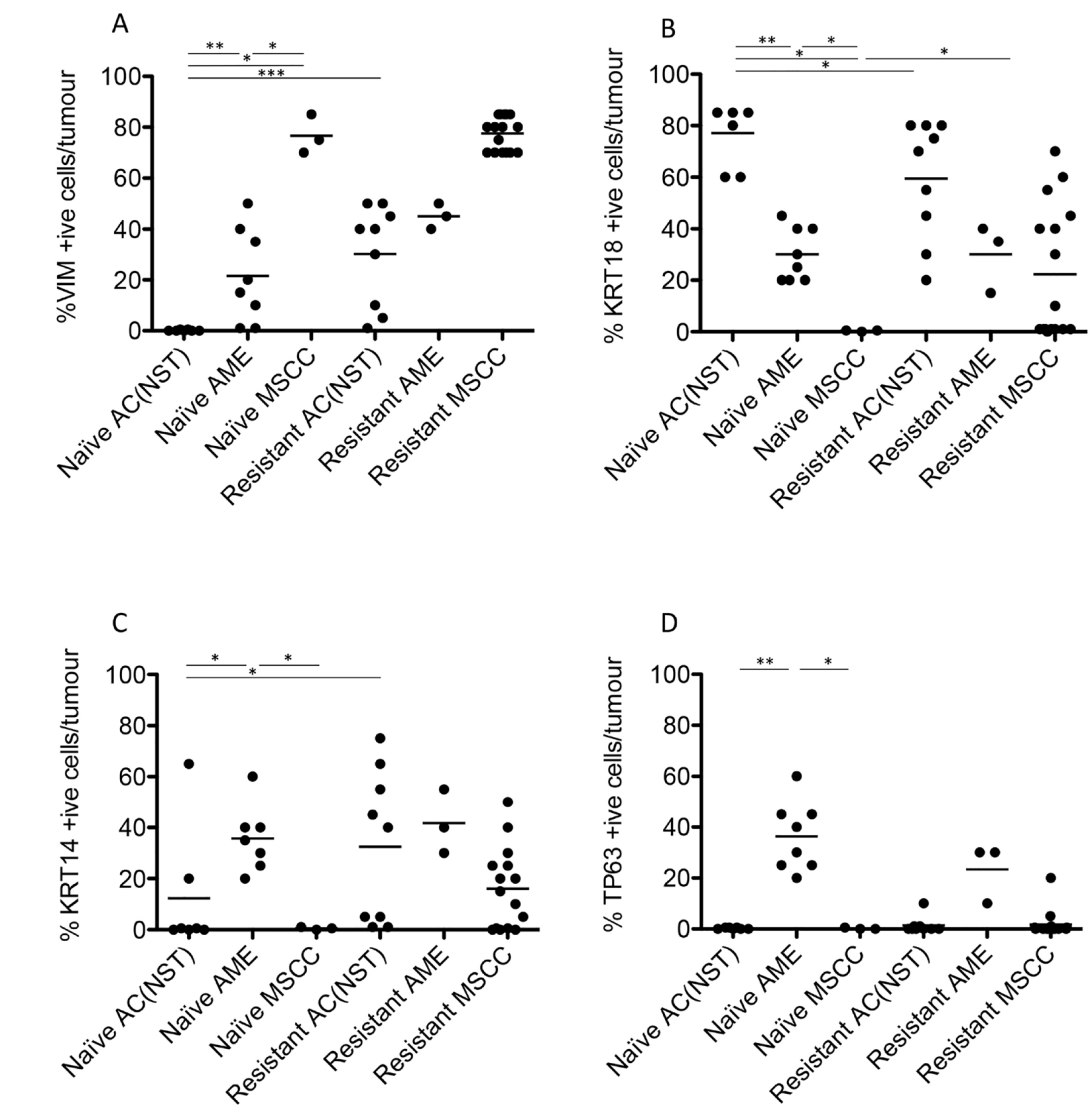
Quantitation of immunohistochemical staining in olaparib-naïve and olaparib-resistant *Brca2*/*p53*-mutant tumours VIM (**A**), KRT18 (**B**), KRT14 (**C**) and TP63 (**D**) positive cells as a percentage of total tumour epithelial cells ^*^p < 0.05, ^**^p < 0.01, Mann Whitney *U* test. Bars represent the mean.

We have previously shown that mammary tumours in a *Brca2*-mutant mouse model generally respond well to olaparib, but that eventually tumours relapse on treatment [3]. To begin to identify possible relevant mechanisms that drive resistance, we first carried out a detailed histopathological analysis of olaparib-naïve and relapsed/resistant tumours from the *BlgCre Brca2/Tp53-*mutant mouse mammary model, based on H&E appearance and immunohistochemical staining for KRT14, KRT18, TP63 and VIM and using our previous histotype classification system [14, 15] (Tables 1 and 2; Figures 1–4).

**Table 1:** Antigen expression patterns in normal mammary epithelium and mesenchymal cells [41, 45–47]

Antigen		Normal mammary epithelial distribution	Mesenchymal cell marker?
KRT18	Keratin 18	Luminal epithelial cells (cytoplasmic)	No
KRT14	Keratin 14	Basal/myoepithelial cells (cytoplasmic)	No
TP63	Tumour protein 63	Basal/myoepithelial cells (nuclear)	No
ESR1α	Estrogen receptor	Luminal epithelial cells (cytoplasmic)	No
CDH1	E-cadhern	Luminal epithelial cells (cell membrane)	No
TWIST1	Twist-related protein 1	Basal/myoepithelial cells	Yes
Ki67	Proliferation marker protein Ki67	Proliferating cells (nuclear)	Proliferating cells (nuclear)
VIM	Vimentin	Basal/myoepithelial cells	Yes
SNAI2	Zinc finger protein Snai2	N/K	Yes
ZEB1	Zinc finder E-box binding homeobox 1	N/K	Yes
ZEB2	Zinc finder E-box binding homeobox 2	Basal mammary stem cells	Yes

**Table 2:** Summary of immunohistochemical staining patterns for KRT18, KRT14, TP63 and VIM in olaparib-naïve and olaparib–resistant tumours

Tumour type	Treatment	% KRT18+ cells	% KRT14+ cells	% TP63+ cells	% VIM+ cells
AC(NST) (*n* = 7)	Naïve	60–85	0–80	<1	<1
AME (*n* = 8)	Naïve	20–45	1–60	20–60	1–50
MSCC (*n* = 3)	Naïve	<1	<1	0	70–85
ASQC (*n* = 2)	Naïve	1–5	50	50–55	<1
AC(NST) (*n* = 9)	Resistant	20–80	1–75	0–10	1–50
AME (*n* = 3)	Resistant	15–40	30–55	10–30	40–50
MSCC (*n* = 15)	Resistant	1–70	0–40	0–5	70–85
ASQC (*n* = 1)	Resistant	<1	60	40	30

AC(NST), adenocarcinoma of no special type; AME, adenomyoepithelioma; MSCC, metaplastic spindle cell carcinoma; ASQC, adenosquamous carcinoma.

**Figure 5:**
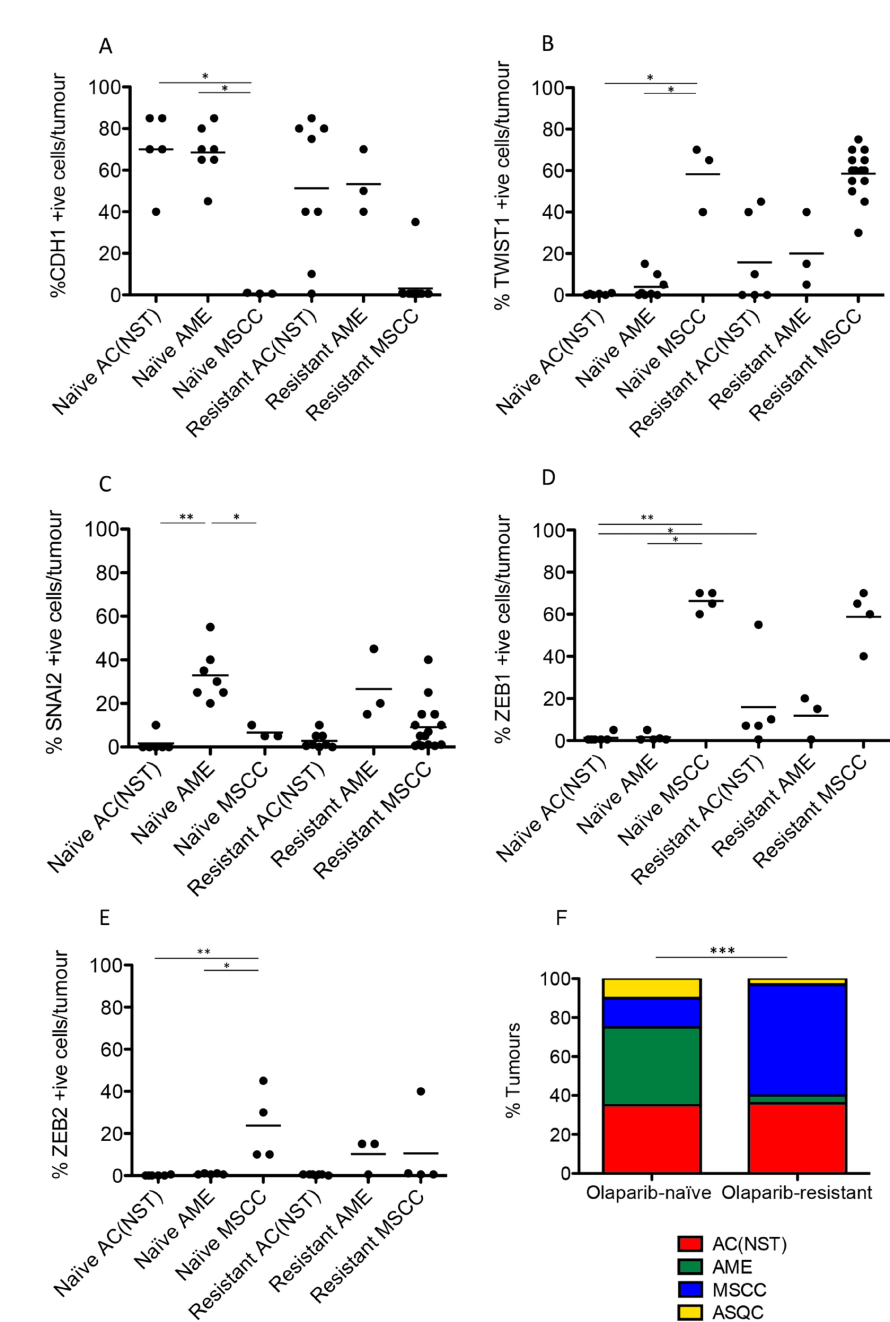
Olaparib-resistant *Brca2*/*p53*-mutant tumours are enriched for an EMT histotype CDH1/E-Cadherin (**A**), TWIST (**B**), SLUG (**C**), ZEB1 (**D**) and ZEB2 (**E**) positive cells as a percentage of total tumour epithelial cells. ^*^p < 0.05, ^**^p < 0.01, Mann Whitney *U* test. Bars represent the mean. **(F)** Proportions of the 4 different tumour types in both olaparib-naïve (*n* = 20) and olaparib-resistant (*n* = 28) tumour cohorts; ^***^p < 0.001, Chi^2^ test.

Consistent with our previous studies [15], assessment of histopathological phenotypes in a cohort of 20 naïve tumours classified seven as adenocarcinoma/invasive ductal carcinoma of no special type (AC/IDC-NST, hereafter AC(NST), 35%), eight as adenomyoepithelioma (AME, 40%), two as adenosquamous carcinoma (ASQC, 10%) and three as carcinosarcoma/metaplastic spindle cell carcinoma (MSCC, 15%). All tumours were ESR1α-negative, although ESR1α-positive cells could be observed in normal epithelial ducts (not shown). In addition to a high percentage of VIM-positive tumour cells, MSCCs had a low percentage of CDH1-positive cells and a high percentage of TWIST1-, ZEB1- and ZEB2-positive cells, characteristic of EMT (Figure 5A–5E).

### Resistance to olaparib is characterised by epithelial–mesenchymal transition

Next, we compared the phenotypes of the 20 naïve tumours versus 28 tumours that initially responded but then relapsed (relapsed/resistant tumours). We observed a significant change in the proportions of tumour phenotypes. MSCCs, that composed just 15% of the naïve tumours, accounted for ∼60% in the relapsed cohort. On the contrary, AMEs were 40% of naïve tumors, versus 6% of relapsed tumours (Table 2; Figure 5F, *p* < 0.001). Furthermore, relapsed AC(NST)s had significantly more VIM-positive cells than treatment-naïve tumours of a similar phenotype, which a similar trend was seen in AMEs (although this did not reach significance; Table 2, Figures 1 and 4A). Interestingly, relapsed MSCCs had increased KRT18-positive cells relative to treatment-naïve MSCCs (Table 2, Figures 1, 2 and 4B). Some relapsed MSCCs contained small regions with histopathological features that resembled AC(NST) or AME, contributing to the increased KRT18-positivity in these tumours, but some spindle cells also showed KRT18 staining. It is not possible to determine if these findings in relapsed MSCCs are due to pre-existing fusiform cells upregulating KRT18 in response to therapy, or whether these KRT18-positive fusiform cells are in fact epithelial cells which have undergone a partial EMT and retained some epithelial marker expression.

Immunohistochemical analysis of the EMT-associated transcription factors ZEB1, ZEB2, TWIST1 and SNAIL2 in relapsed AC(NST)s (Figure 5B–5E) demonstrated a significant increase in ZEB1 staining (*p =* 0.03). Moreover, three out of six tumours analyzed showed increased TWIST1 staining compared to naïve AC(NST)s (although there were no differences in SNAI2 or ZEB2 staining). Resistant MSCCs and AMEs showed no significant differences in ZEB1 or TWIST1 compared to naïve tumours (Figure 5).

### Olaparib-responsive epithelial tumours express VIM

**Figure 6:**
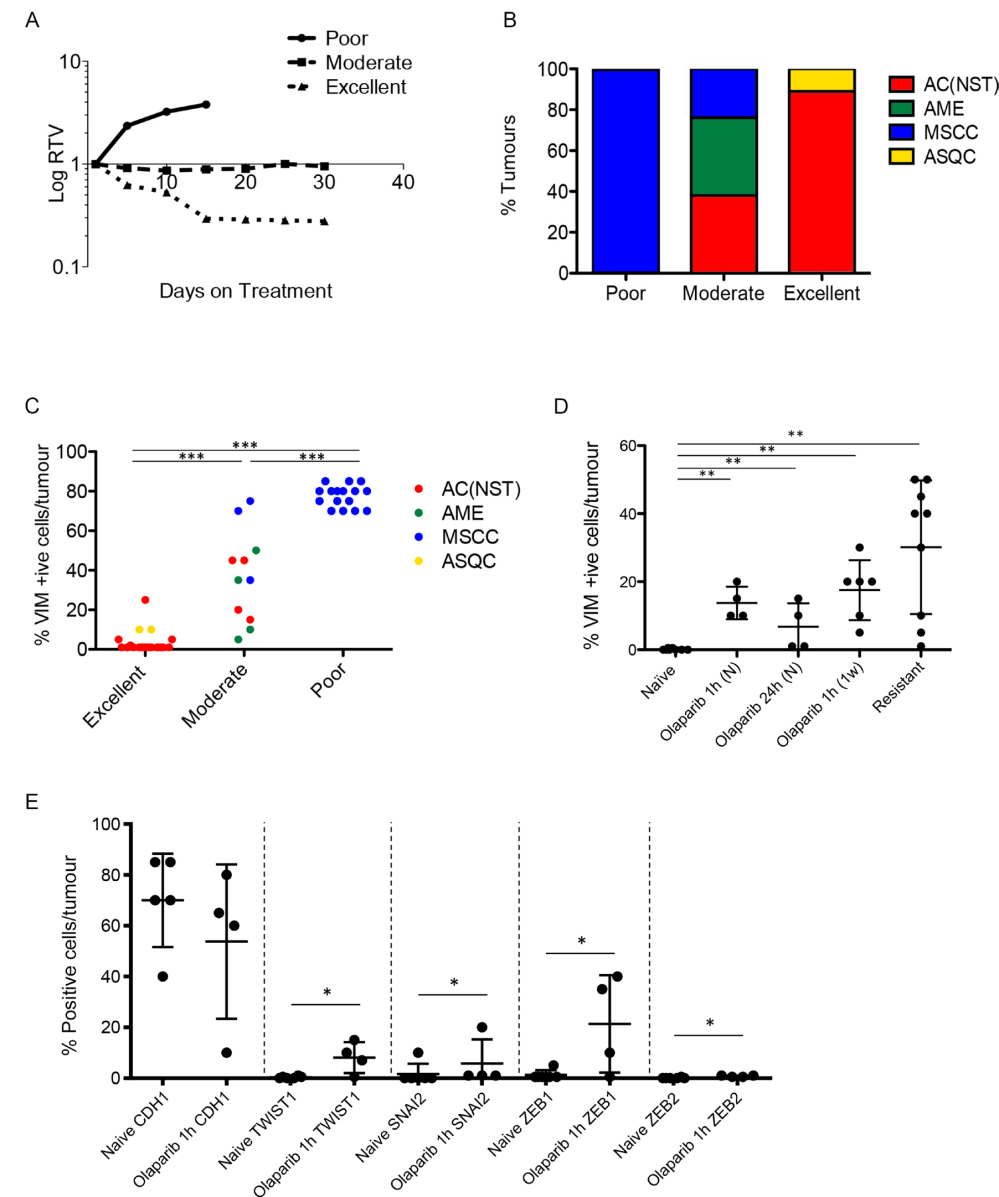
Olaparib response is correlated with tumour phenotype (**A**) Illustrative graph showing examples of relative tumour volume (RTV) plots representing poor, moderate and excellent responders. (**B**) Proportions of the 4 different tumour types in poor (*n* = 16), moderate (*n* = 13) and excellent (*n* = 18) responder cohorts. **(C)** VIM expression in different olaparib response groups. Tumours were harvested 1 hour after their final dose of olaparib. ^***^p < 0.001, Mann Whitney *U* test. (**D**) VIM expression in AC(NST)s at various stages of treatment with olaparib. Tumours were harvested from olaparib-naïve mice (Naïve, *n =* 8), mice given a single dose (1 hour and 24 hour, *n =* 4 per cohort), mice treated daily for one week (1 week, *n =* 6), mice whose tumours were responding excellently or moderately (excellent responder; *n =* 14, or moderate responder; *n =* 5) and mice in which tumours had become resistant (resistant, *n =* 9). All tumours were harvested 1 hour after mice were treated with their final dose, except in naïve and 24 hour. ^**^p < 0.01, ^***^p < 0.001, Mann Whitney *U* test. Black line represents the mean. (**E**) Additional EMT markers (CDH1; TWIST, SNAI2; ZEB1; ZEB2) in AC(NST)s comparing olaparib-naïve tumours and tumours that have seen a single dose of olaparib and taken 1 hour later. ^*^p < 0.05. Note that for ZEB2, five of the six naïve tumours analysed showed no staining at all, while one tumour had staining in <1% of cells. In contrast, all four olaparib-dosed tumours showed staining, although only in up to 1% of cells. This was sufficient to result in a significant difference between the groups.

While extended olaparib treatment in this model inevitably results in resistance of tumours to therapy and relapse, the initial response to treatment was heterogeneous. Tumour response to olaparib over the first 30 days of treatment could be classified as: excellent responders, which show a decrease in size; moderate responders, which stop growing but do not decrease substantially in size; and poor responders (including non-responders) which continue to grow similarly to the no-drug control (Figure 6A). Most tumours showed at least a moderate response, with poor responders being rare.

To strengthen the correlation between EMT and resistance, and determine whether tumour histopathology correlated with response categories, a cohort of 47 tumours were analyzed in mice which received daily IP 100 mg/kg olaparib and were culled around 30 days into treatment. 16 tumours from this cohort were classed as poor responders, 13 as moderate responders and 18 as good responders.

Assessment of all 16 poorly-responding tumours revealed that they were exclusively MSCCs (Figure 6B), and were histologically identical to the naïve MSCCs we had previously analysed. The moderate responder tumors were 38.5% AMEs, 38.5% AC(NST)s and 23% MSCCs (Figure 6B). Notably, compared to naïve AC(NST)s, moderately-responding AC(NST)s showed a higher percentage of VIM-positive epithelial cells (18–45%; Figure 6C). Moderately-responding MSCCs, in contrast to naïve or poor responders, were not composed exclusively of fusiform spindle cells; rather, each contained regions of cells with epithelial morphology with immunostaining patterns similar to naïve AC(NST)s. Moderately-responding AMEs showed similar staining to naïve AMEs, with the exception of KRT18 staining which was present in less than 1% of tumour cells (compared to 20–45% positive cells in naïve tumours; *n* = 8). The excellent responders were predominantly AC(NST)s (89%) with the remaining 11% ASQCs (Figure 6B).

Immunohistochemical staining of excellent responders showed similarities to naïve tumours, with the exception that AC(NST)s showed VIM positivity in up to 25% of epithelial-like tumour cells. However, excellent responders had a significantly lower percentage of VIM staining than moderate responders (Figure 6C, *p* < 0.01). We assessed whether percentage of VIM positivity in the three responding groups correlated with response to olaparib independently of tumour type. In the tumour cells of the excellent responders we observed a significantly lower percentage of positive VIM staining compared to moderate or poor responders. In parallel, the moderate responders showed a lower percentage of VIM-positivity than the poor responders (Figure 6C), overall indicating that the percentage of VIM positivity is inversely proportional to the efficacy of the response to olaparib.

To strengthen the correlation, and determine how soon/quickly VIM expression was induced after the olaparib treatment started, naïve AC(NST) tumours from mice treated for 1 hour, 24 hours or 1 week were analysed (Figure 6D). Remarkably, we observed a mean incidence of ∼15% in VIM-stained cells 1 hour post first dose (*n* = 4), but 24 hours after a single dose this had fallen back to a mean of 6.75% (range 1–15%; *n* = 4). After a 1-week regimen, ∼25% of cells in previously treatment-naïve tumours were VIM positive. Therefore, expression of this key marker of EMT was rapid, drug-dependent and maintained with extended treatment. Furthermore, when we analysed additional markers of EMT olaparib (CDH1; TWIST; SNAI2; ZEB1; ZEB2) in AC(NST) tumours after a single dose of olaparib, we found a significant increase in the proportion of cells staining for TWIST, SNAI2, ZEB1 and ZEB2 (*p =* 0.033, *p =* 0.043, *p =* 0.033 and *p =* 0.014 respectively) (Figure 6E).

In summary, these findings indicate that not only is innate olaparib resistance associated with EMT in our model, but that acquired resistance is also correlated with either mesenchymal-like tumour phenotype or, in those tumours that retain a more epithelial-like appearance, the rapid treatment-dependent up-regulation of a subset of EMT markers.

### Upregulation of P-gp is not a mechanism of resistance to olaparib in the *Brca2*-mutant model

**Figure 7:**
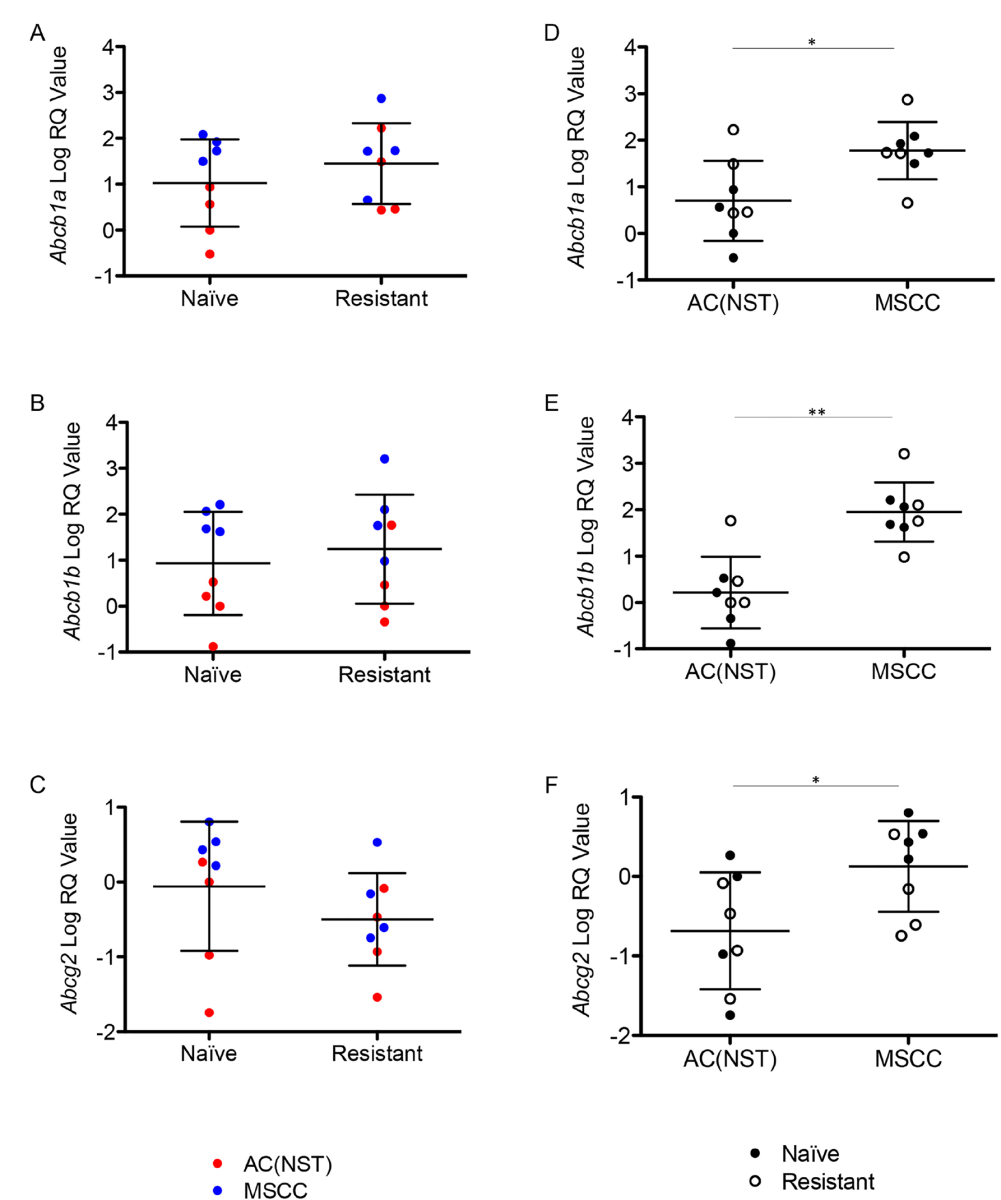
Up-regulation of P-gps is correlated with tumour phenotype (**A**–**C**) Expression of *Abcb1a* (**A**) *Abcb1b* (**B**) and *Abcg2* (**C**) in olaparib-naïve tumours (Naïve) compared to olaparib-resistant tumours (Resistant), independent of tumour phenotype. Filled or empty markers represent olaparib-naïve or –resistant tumours respectively. (**D**–**F**) *Abcb1a* (**D**) *Abcb1b* (**E**) and *Abcg2* (**F**) expression by qRT-PCR in MSCCs compared to AC(NST)s, independent of treatment. ^*^p < 0.05, ^**^p < 0.01, Mann Whitney *U* test. Error bars represent standard deviation.

Innate resistance to olaparib in a *K14Cre Brca2/Tp53-*mutant mouse model showed a link between EMT-like tumours and high expression of P-gp in a previous study [8]. We also previously demonstrated that the majority of olaparib-resistant tumours in the *BlgCre Brca2/Tp53*-mutant mammary tumour model had increased expression of one or more P-gp [3]. To test whether in the olaparib-resistant cohort there is a link between the increase in proportion of MSCC/EMT-like tumours and the up-regulation of the P-gp *Abcb1a*, *Abcb1b* and *Abcg2* [3], we compared their expression in olaparib-naïve and olaparib-resistant tumours. Overall, we did not observe significant changes in the expression levels of any relevant P-gp between the naïve and resistant tumours (Figure 7A–7C). Strikingly, comparison between tumour phenotypes showed that MSCCs had significantly higher expression of all three receptors compared to AC(NST)s (Figure 7D–7F, *p =* 0.015, *p* < 0.01 and *p =* 0.038 respectively). This observation was not dependent of treatment, suggesting that a high P-gp level is likely to be a characteristic of MSCCs in this model. Therefore, the high levels of P-gp previously observed in olaparib-resistant tumours [3] are likely due to enrichment for MSCCs.

**Figure 8:**
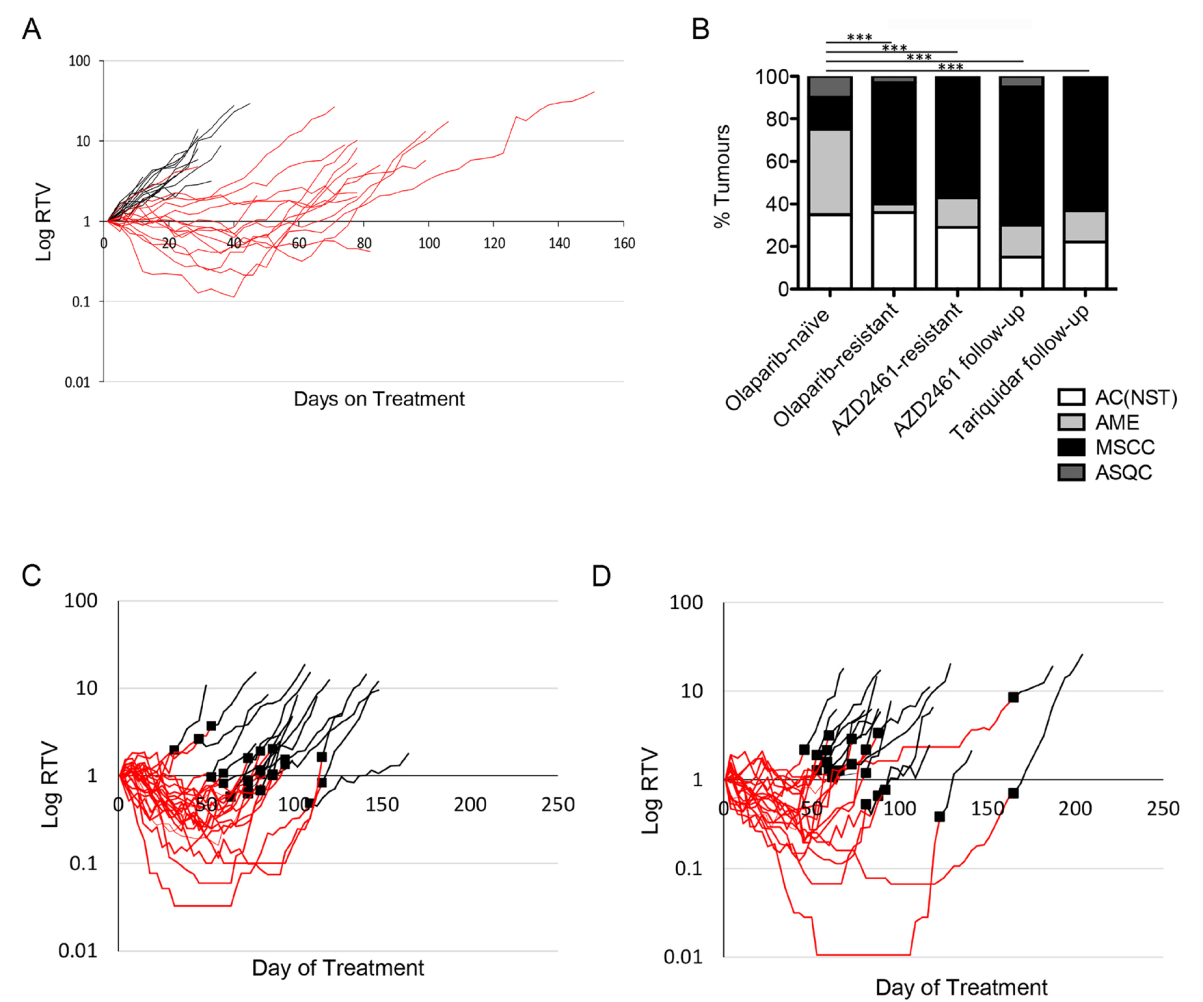
*Brca2* mammary tumours acquire resistance to AZD2461 (**A**) Relative Tumour Volume (RTV) plot for AZD2461 monotherapy administered to *BlgCre Brca2/Tp53-*mutant mouse mammary model. Each line represents an individual tumour, with an RTV of 1 representing tumour size at start of treatment. Black lines are tumours from mice treated daily with vehicle (*n =* 20), red lines tumours from mice treated daily with 100mg/kg AZD2461 (*n =* 20). (**B**) Comparison of tumour types between olaparib-naïve tumours (*n =* 20) and those that became olaparib-resistant (*n =* 20), resistant to AZD2461 monotherapy (AZD2461-resistant, *n =* 14), resistant to olaparib then treated with AZD2461 follow-up therapy (AZD2461 follow-up, *n =* 20) and resistant to olaparib then treated with the Tariquidar/olaparib combination follow-up therapy (Tariquidar follow-up, *n =* 27). ^***^p < 0.001, Chi-squared test. (**C**–**D**) RTV plots of AZD2461 follow-up (**C**) and Tariquidar (**D**) therapy. Tumours which developed resistance during daily treatment with 100 mg/kg olaparib (red lines) did not respond to follow-up with either daily 100 mg/kg AZD2461 (**C**, black lines, *n =* 23) or combination treatment of 2 mg/kg Tariquidar with 100 mg/kg olaparib (**D**, black lines, *n =* 20). Black squares denote the day of switchover from one treatment to the other.

To shed further light on the functional correlation between P-gp upregulation, EMT and PARPi-resistance, we treated our mouse models with an alternative PARP inhibitor AZD2461, which is not a substrate for P-gp [16, 17]. Consistent with the published literature, we first demonstrated that AZD2461 is as effective as olaparib in our PARPi-naïve tumours (Figure 8A), and that resistant tumours develop on treatment [17], so in these tumours at least, P-gp levels are unlikely to be a direct mechanism of drug resistance. Interestingly, these AZD2461-resistant tumours were also enriched for MSCCs (Figure 8B). In a follow up experiment, tumour-bearing mice were first treated with daily olaparib; then, when tumours became resistant, were switched to AZD2461. As shown in Figure 8C, none of these tumours regressed, suggesting that if there was a resistance mechanism caused by P-gp upregulation, this could not be reversed by a drug that is not a P-gp substrate. Furthermore, the spectrum of tumour phenotypes was similar to olaparib-resistant tumours (Figure 8B). Hence, in this scenario, upregulation of P-gp also does not seem to be the direct cause of resistance/relapse.

We also tested the P-gp inhibitor tariquidar as a follow-up therapy on olaparib-resistant tumours in this model, using daily IP 2mg/kg Tariquidar 30 minutes prior to olaparib dosing. Similarly, we saw no differences in the growth curves compared to olaparib, in any of the 20 tumours tested (Figure 8D). Again, the resistant tumours were enriched for MSCCs (Figure 8B). The overall conclusion from these two independent lines of experiments is that over-expression of P-gp is associated with EMT features in resistant tumours in this model but is not the major mechanism of resistance to olaparib.

### Resistant tumours accumulate olaparib and have reduced PAR levels

**Figure 9:**
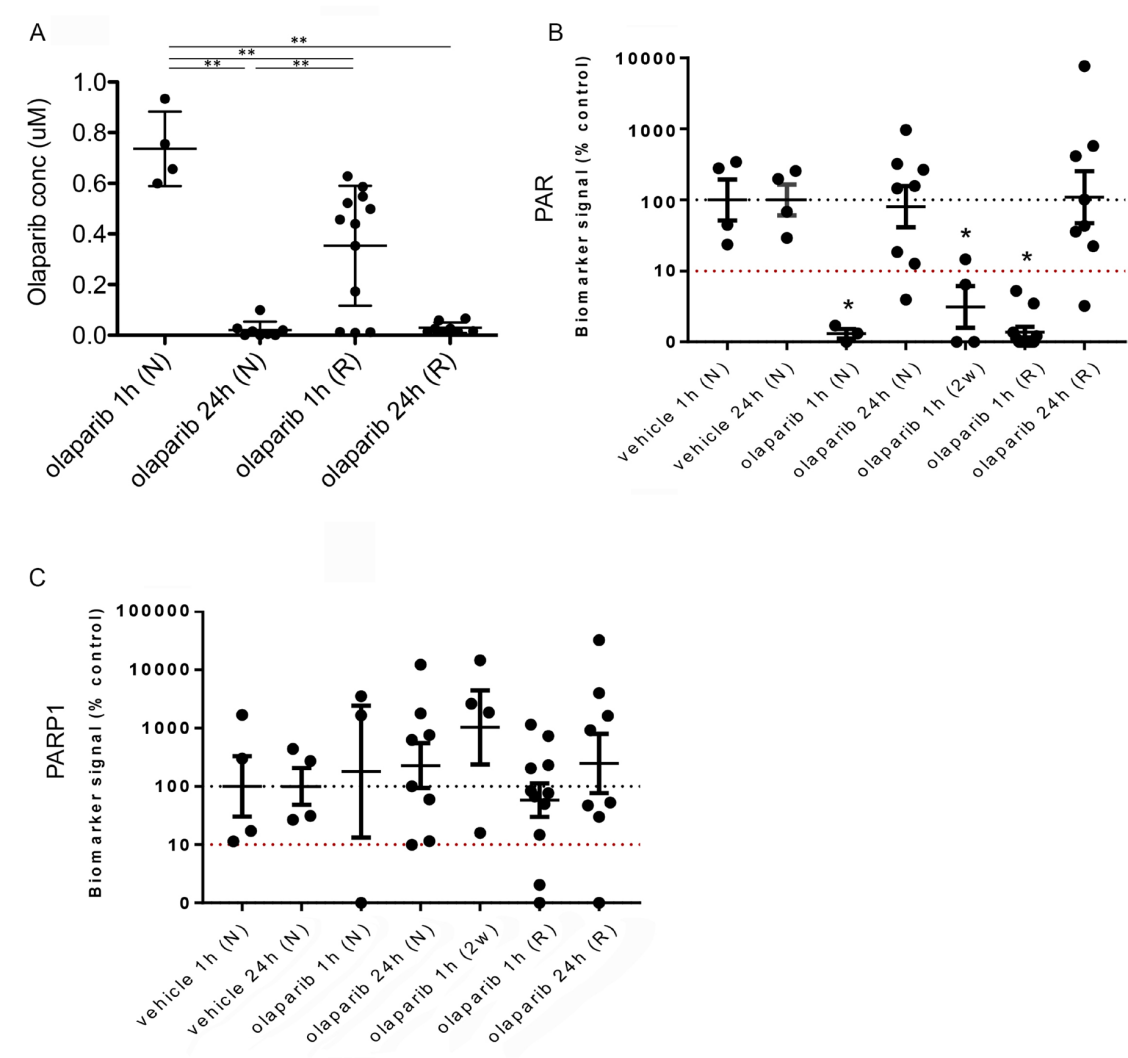
Reduced olaparib concentration in olaparib-resistant tumours does not correlate with increased PAR levels (**A**) Olaparib concentrations in tumours from mice treated with either a single dose of olaparib and taken either 1 hour (olaparib 1 h (N)) or 24 hours (olaparib 24 h (N)) later, or olaparib-resistant tumours taken either 1 hour (olaparib 1 h (R)) or 24 hours (olaparib 24 h (R)) after the final dose. ^**^p < 0.01, Mann Whitney *U* test. Error bars represent standard deviation. (**B**, **C**) Quantitation of western blots performed to analyse protein levels of PAR (**B**) and PARP-1 (**C**) in tumours from mice treated with a single dose of vehicle and taken either 1 hour (vehicle 1 h (N), *n =* 4) or 24 hours (vehicle 24 h (N), *n =* 4) later, tumours from mice treated with either a single dose of olaparib and taken either 1 hour (olaparib 1 h (N), *n =* 3) or 24 hours (olaparib 24 h (N), *n =* 8) later, tumours from mice treated for 2 weeks with daily olaparib and taken 1 hour after the final dose (olaparib 1 h (2w), *n =* 4) or olaparib-resistant tumours taken either 1 hour (olaparib 1 h (R), *n =* 11) or 24 hours (olaparib 24 h (R), *n =* 8) after the final dose. Vinculin was used as an endogenous control. ^*^p ≤ 0.05, ANOVA.

Next, to determine whether resistance to PARP inhibitors might be explained by pharmacokinetic/pharmacodynamic (PK/PD) reasons, and whether this might correlate with the increase in mesenchymal features in resistant tumours, we analyzed olaparib concentration in tumours from four cohorts of mice (Figure 9A): olaparib-naïve mice, taken 1 or 24 hours after a single dose of 100 mg/kg olaparib, and those in which tumours had become resistant to olaparib, taken 1 or 24 hours after a final dose of IP 100 mg/kg olaparib.

Olaparib concentrations in naïve tumours one hour after a single dose ranged from 0.6–0.93 µM, whilst those 24 hours after were significantly lower (<0.1 µM, *p* < 0.01). Olaparib concentrations in resistant tumours one hour after their final dose were significantly lower than in naïve tumours one hour after a single dose (resistant tumours = 0.01–0.628 µM; naïve tumours = 0.6–0.93 µM; *p* < 0.01) but significantly higher than in naïve tumours taken 24 hours after a single dose (resistant tumours = 0.01–0.628 µM; naïve tumours = <0.1 µM; *p* < 0.01). Olaparib concentrations in resistant tumours 24 hours after their final dose were similar to those in naïve tumours taken 24 hours after a single dose (resistant tumours ≤0.015–0.16 µM; naïve tumours ≤0.1 µM).

We next analyzed the pharmacodynamics of PAR levels in the naïve and resistant tumours, as well as in those from mice either 1 or 24 hours after a single dose of vehicle and tumours that were responding to a 2-week daily dose of IP 100 mg/kg olaparib, and taken 1 hour after their final dose (Figure 9B). Both vehicle cohorts showed similar PAR levels and did not differ significantly. Tumours from naïve mice 1 hour after a single dose of olaparib showed reduced PAR levels compared to those treated with vehicle, correlating with the high olaparib concentration seen in these tumours (*p =* 0.053). Naïve-tumours taken 24 hours after a single dose of olaparib showed similar PAR levels to those seen in the vehicle-treated tumours. The 2-week olaparib treatment cohort also showed reduced PAR levels compared to vehicle (*p =* 0.021), correlating with these tumours responding to PARP inhibitor treatment.

Importantly, PAR levels in resistant tumours taken 1 hour after their final dose were significantly lower compared to the vehicle groups (*p =* 0.024), independent of tumour type. Indeed, levels were similar to those seen in naïve tumours 1 hour after a single dose, despite olaparib levels in the resistant tumours being lower than those in the naïve tumours. PAR levels in resistant tumours taken 24 hours after their final dose were not significantly different to those treated with vehicle.

These findings suggest that either a sufficient concentration of olaparib can be achieved in resistant tumours for efficacious inhibition of PARP1, or, that the resistant tumours in fact have low levels of PARP1 expression. As olaparib is thought to have its effect by trapping PARP-1 at replicating forks and triggering their collapse, this would indicate a potential mechanism of resistance. To investigate this, we analyzed PARP1 levels (Figure 9C). Expression levels were highly variable across the cohorts, but there were no significant differences between naïve and resistant tumour cohorts, and no evidence to suggest that low PAR levels in resistant tumours correlated with lower expression of PARP1.

Therefore, PARP1 is expressed in olaparib-resistant tumours and sufficient olaparib concentrations can be achieved in resistant tumours to suppress its PARylation activity. However, it is not clear if the concentration in resistant tumours is sufficient for PARP trapping.

### Olaparib-resistant *Brca2* tumours remain defective for homologous recombination-directed DNA repair

PARP-1 inhibition in BRCA-defective tumours is expected to cause unrepaired double-stranded breaks in DNA [1, 2]. In our model, given that the PK/PD and PARP-1 expression data demonstrate that in olaparib-resistant tumours PARP-1 is still expressed and its enzymatic activity is being inhibited, these tumours may be able to either suppress the DNA damage following PARP-1 inhibition, or repair the damage by re-activating homologous recombination-directed repair (as reported in other studies, although unlikely here considering the nature of the *Brca2* recombined allele used in the study) [10], or alternatively tolerate the damage via an as-yet unidentified mechanism.

**Figure 10:**
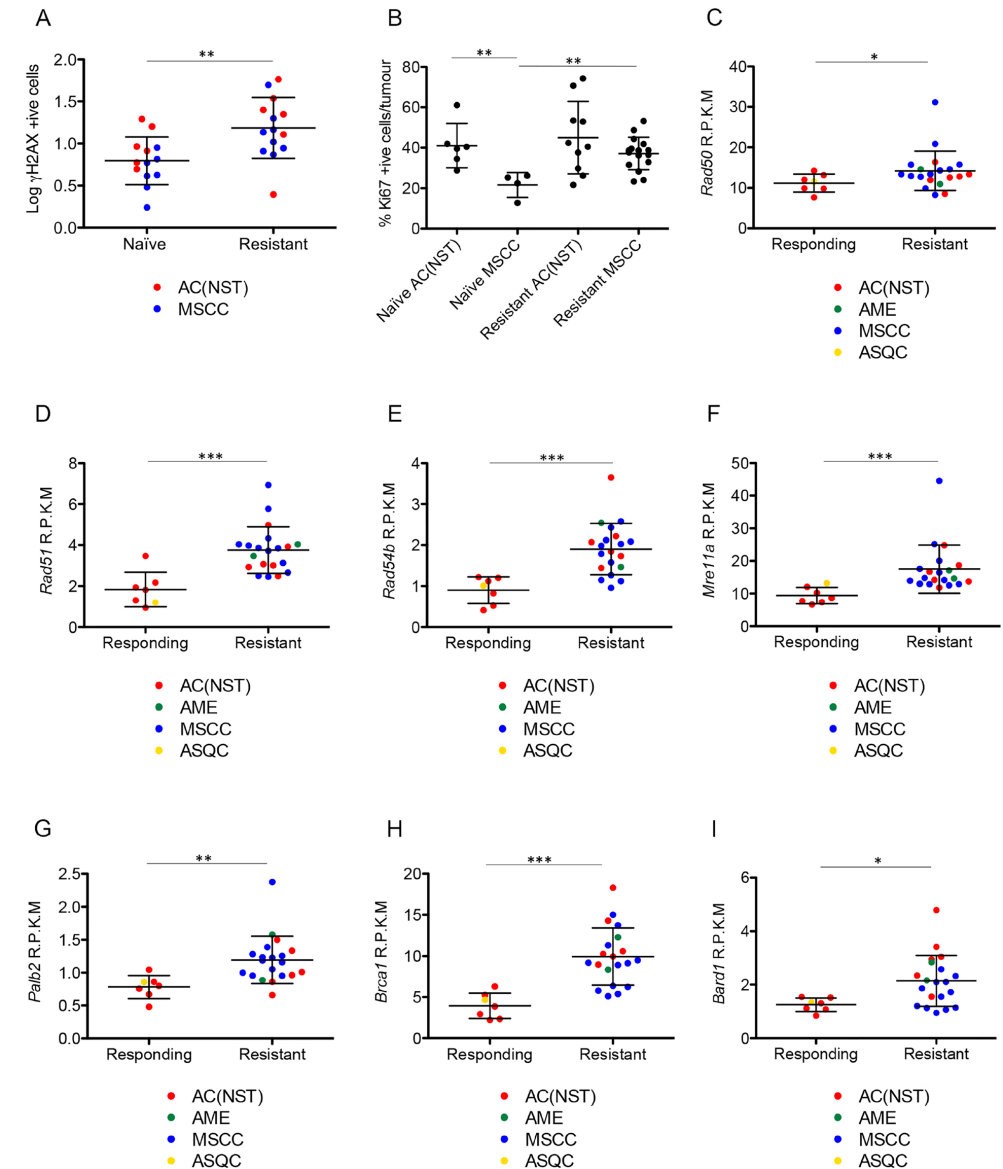
Olaparib-resistant tumours have DNA damage and show elevated expression of HR-associated genes (**A**) γH2AX positive cells as a percentage of total tumour cells in olaparib-naïve compared to olaparib-resistant tumours, independent of tumour type. ^**^p < 0.01, Mann Whitney *U* test. Error bars represent standard deviation. (**B**) Ki67 levels in olaparib-naïve AC(NST)s and MSCCs compared to olaparib resistant tumours of the same phenotypes. ^**^p < 0.01, Mann Whitney *U* test. Error bars represent standard deviation. **(C–I)** Expression levels of *Rad50* (**C**), *Rad51* (**D**), *Rad54b* (**E**), *Mre11a* (**F**), *Palb2* (**G**), *Brca1* (**H**) and *Bard1* (**I**) comparing tumours that were decreasing in size in response to olaparib therapy (Responding) and tumours that became resistant to olaparib therapy (Resistant), analysed by RNA-seq. R.P.K.M = reads per kilobase of transcript per million mapped reads. ^*^p < 0.05, ^**^p < 0.01, ^***^p < 0.001 Mann Whitney *U* test. Error bars represent standard deviation.

To address these possibilities, we first analyzed levels of DNA damage in olaparib-resistant tumours by assessing numbers of γH2AX positive cells. Olaparib-resistant tumours showed a significant increase in the percentage of γH2AX positive cells compared to olaparib-naïve tumours (Figure 10A, *p =* 0.006). Comparison of Ki67 levels between naïve and resistant tumours showed no significant difference for AC(NST)s but a significant increase for MSCCs (Figure 10B, *p* < 0.01), showing that although resistant tumours have increased DNA damage, the cells are still able to proliferate, particularly in MSCCs.

**Figure 11:**
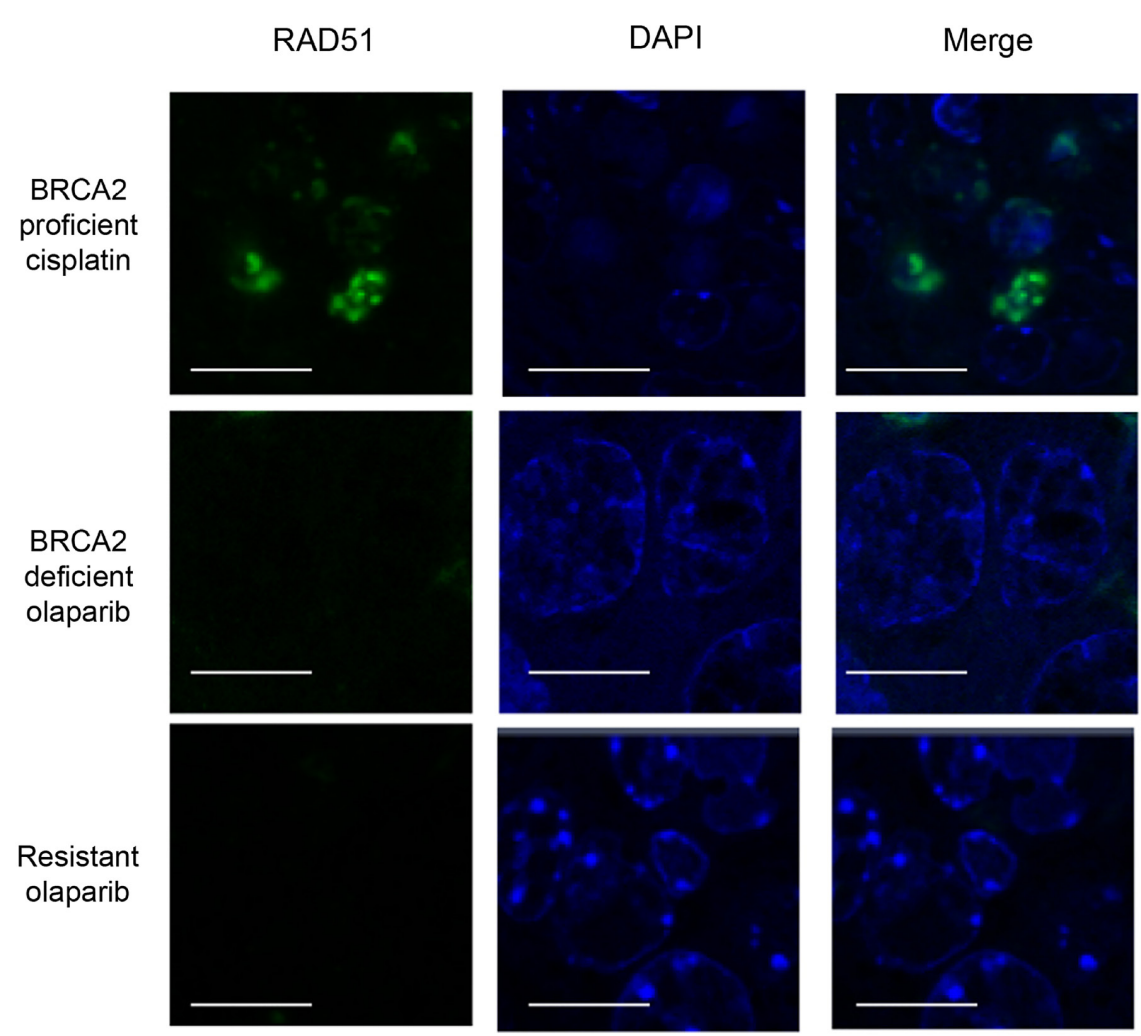
Olaparib-resistant tumours do not have Rad51 foci Rad51 staining in intestinal cells of a BRCA2 wild-type mouse taken 24 hours after a single dose of 10 mg/kg cisplatin (BRCA2 proficient cisplatin) and either a mammary tumour from a mouse treated with a single dose of olaparib and taken 24 hours later (BRCA2 deficient olaparib) or a olaparib-resistant tumour taken 24 hours after the final dose (Resistant olaparib) from our *Brca2/p53*-mutant mammary tumour model. Scale bars represent 10 µm. Pictures taken with Zeiss LSM710 confocal microscope using a 60× objective.

Having shown that DNA damage is increased in olaparib-resistant tumours, we next analysed whether HRR could have been restored to repair this damage [7, 9, 18], although unlikely in our model, given the permanent nature of deletion of the *Brca2* conditional allele. We analyzed the mRNA expression levels of components of the HR pathway using RNAseq. RNA was analyzed from tumours that were responding well to olaparib (responding; *n* = 7) or that had responded well initially but had become resistant and were at least 5 times larger than their smallest size (resistant; *n* = 22). All mice had received daily IP 100 mg/kg olaparib and were culled 1 hour after their final dose. Histopathological analysis showed that the responding cohort consisted of six AC(NST)s and one ASQC, whilst the resistant cohort comprised 13 MSCCs, seven AC(NST)s and two AMEs. Both cohorts showed the same IHC staining patterns as other excellent responders and resistant tumours, as described above. *Rad50*, *Rad51*, *Rad54b*, *Mre11a*, *Palb2*, *Brca1* and *Bard1* all showed significantly higher expression in resistant tumours compared to responding tumours (Figure 10C–10I, *p =* 0.05, *p* < 0.001, *p* < 0.001, *p* < 0.001, *p =* 0.002, *p* < 0.001 and *p =* 0.012 respectively). Importantly, however, olaparib-resistant tumours taken 24 hours after their final dose were negative for RAD51 foci (Figure 11), irrespective of tumour phenotype, confirming that despite components of the pathway being up-regulated, the HRR pathway is compromised in these *Brca2* knockout tumours, as expected.

## DISCUSSION

PARP inhibitors have shown great promise in clinical trials and are likely to come into general use in HR-deficient (and in particular BRCA1/2 loss of function) cancers [19, 20]. However, both primary and acquired resistance has been seen in the clinic, and in pre-clinical models [3, 4, 7], and a number of mechanisms have been proposed. These include up-regulation of P-gps [4], restoration of BRCA function by genomic rearrangements [7, 10], loss of TP53BP1 [9] and mutations in *PARP-1* [11]. Here we investigated resistance to olaparib in our *BlgCre Brca2/Tp53-*mutant mouse model.

The most striking feature of olaparib-resistant tumours was the increase in proportion of MSCCs compared to olaparib-naïve tumours, together with the partial acquisition of EMT characteristics, such as increased VIM and TWIST1, in resistant tumours that had not fully acquired the MSCC phenotype. EMT has been shown to correlate with drug-resistance in pancreatic cancer [21, 22], urothelial cancer [23] and non-small cell lung carcinomas [24], and with resistance to a number of classes of drugs including cisplatin [25, 26], cyclosporine A [27] and adriamycin [28], suggesting that EMT conversion is a generic response by tumour cells to toxic stresses. Furthermore, previous studies have suggested a link between EMT and metabolic reprogramming. During EMT cells undergo loss of matrix attachment and in mammary epithelial cells overexpressing the ERBB2 oncogene this has been shown to regulate metabolic activity [29]. Metabolic reprogramming as a survival strategy in different environments has been demonstrated in tumour cells [30–32] and if cells undergoing EMT reprogram their metabolic ability this could also contribute to acquired resistance.

Innate resistance of MSCC-like tumours to multiple drugs, including olaparib, has been previously shown in a *K14Cre Brca2/Tp53*-mutant mouse model [8] and this was linked to P-gps. P-gps are transmembrane protein pumps belonging to the family of multi-drug resistance proteins that are critical in the resistance to a number of drugs (reviewed in [33]). The *K14Cre Brca2/Tp53*-mutant mouse model study suggested that the up-regulation of these genes contributed to innate resistance, also showing that the addition of the P-gp-inhibitor Tariquidar sensitized MSCC-like tumours that showed high expression of P-gps to the drug therapy [8]. In support of this, we had previously published that the majority of olaparib-resistant tumours in our model show up-regulation of P-gps [3]. However, treatment of animals with olaparib-resistant tumours with either a PARP inhibitor with less affinity for P-gps, or olaparib in combination with the P-gp inhibitor Tariquidar, failed to overcome resistance. Furthermore, AZD2461 monotherapy of naïve tumours did not delay the appearance of resistance. These findings suggest that up-regulation of P-gps is a characteristic of some resistant tumours rather than a mechanism of resistance. Supporting this, analysis of *Abcg2, Abcb1a* and *Abcb1b* showed that ‘up-regulation’ correlated with tumour phenotype rather than olaparib resistance, with all three showing higher expression in MSCCs compared to AC(NST)s, suggesting that the upregulation of these genes noted previously was due to the increase in the proportion of MSCCs in olaparib-resistant cohorts.

Analysis of olaparib concentration and PARP activity in olaparib-resistant tumours showed that while there was a significant reduction in olaparib concentration 1 hour after their final treatment when compared to olaparib-naïve tumours 1 hour after their first treatment, PARP activity was still suppressed. This reduction in intra-tumoural olaparib levels may in fact be due to up-regulation of P-gps, but as this does not rescue PARP-1 PARylation activity it adds further weight to the notion that increased drug efflux is not a mechanism of resistance to olaparib in our model. The differing results seen between our mouse model and the *K14Cre Brca2/Tp53*-mutant mouse model may be due to the differences in cell-of-origin of the tumours, resulting in variances in tumour phenotypes, as we have shown in a previous publication [15]. Our findings also differ from a *Brca1/p53* mutant breast cancer model, where sensitivity to olaparib was at least partially restored upon either follow-up treatment [4, 9]. This suggests that different mechanisms of resistance to PARP inhibition must exist in these genetically distinct mouse models, with important clinical implications if such differences were replicated in *BRCA1-* and *BRCA2-*mutated human breast cancers.

Restoration of HR by secondary mutations in *BRCA2* which restore gene function has been shown to result in resistance to olaparib [7, 10]. In our *BlgCre Brca2/Tp53-*mutant mouse model, staining for γH2AX showed that olaparib-resistant tumours contained high levels of DNA damage, but the absence of nuclear Rad51 focus formation suggests that the HR pathway had not been restored. However, we cannot exclude the possibility that the response kinetics to olaparib treatment may have been altered in the tumours and that restoration of HR function may have been delayed. This caveat could be resolved by future studies addressing responses at additional timepoints after dosing. It also remains formally possible that while the reduction in concentration of olaparib observed in resistant tumours was not sufficient to prevent enzymatic PARylation activity, it was sufficient to reduce PARP trapping below a threshold which permitted tumour survival. However, a clear biological effect of increased γH2AX staining was observed in resistant compared to naïve tumours, which would be consistent with continuing olaparib-mediated DNA damage events.

Therefore, while we have tested a number of hypotheses addressing the underlying resistance mechanism in our *Brca2* loss-of-function mammary tumour model, including upregulation of P-gp, altered PD/PK, loss of expression of the drug target and restoration of HRR, none of these hypotheses can be supported. Indeed, the only strong correlate of either primary resistance or relapse on therapy remains EMT and the rapid expression of EMT-associated markers in a treatment-dependent manner. A recent study in small cell lung cancer identified a link between EMT and resistance to PARP inhibitors in that disease setting [34] and PARP-1 has been shown to regulate EMT in prostate cancer models through regulation of TGFβ signalling and changes in levels of ZEB1 [35]. Therefore, it is likely that some as-yet undefined aspect of the biology of mesenchymal cells is responsible for the PARP-inhibitor resistance phenotype across a variety of tissues. Importantly, we now show the rapidity of this response, making interventions which could block this process or target the underlying mechanism attractive options for olaparib combination therapy.

## MATERIALS AND METHODS

### Animal model and genotyping

All animal procedures were carried out according to current UK Home Office regulations following local ethical committee approval and under the authority of the appropriate personal and project licenses. ARRIVE guidelines were followed.

The *Brca2*-mutant model has previously been described [3]; in brief, these mice carry a *Cre* transgene under the control of the *Blg* promoter, and floxed alleles for *Brca2* and *p53*. Mice were maintained on an outbred, Black 6 (C3H) background and were fed standard diet and water *ad libitum*. Female mice developed mammary tumours from six months of age, with a median of nine months. PCR conditions for the *Blg-Cre* transgene and the *Brca2*^*fl*^ and *p53*^*fl*^ alleles have been previously described [36–38].

### Drug formulations and dosing

Olaparib was prepared as described previously [3] and injected at 100 mg/kg by single bolus injection. AZD2461 was made up at 10 mg/ml in methylcellulose, in a foil-wrapped glass vial with continuous stirring at room temperature, and administered by oral gavage at 100 mg/kg. Tariquidar was diluted to 0.2 mg/ml in 5% dextrose and IP-injected at 2 mg/kg 30 minutes before olaparib.

### Tumour analysis

Tumour size was analysed as described previously [3]. Detailed histopathological analysis was performed blinded to tumour response using previously-established criteria [14, 15] based on human breast tumour classification [39].

### Immunohistochemistry

Immunohistochemistry for KRT18, KRT14, TP63, ESR1α, CDH1, TWIST1 and Ki67 used mouse monoclonal antibodies (see Table 3 for antibody details). VIM was detected using a goat polyclonal antibody and SNAI2, ZEB1 and ZEB2 were detected using rabbit monoclonal antibodies. All stains, other than Ki67, were scored as previously described [14, 15]. Ki67 was scored blind by manually counting the number of positive cells per image using Image J software.

**Table 3:** Details of antibodies used in immunohistochemistry and western blotting and Taqman probes used for qRT-PCR analysis

Antibody clone, dilution and source details			
Antigen	Clone	Dilution	Source
KRT18	65028	1:5	Progen Biotechnik GmbH, Heidelberg, Germany
KRT14	ab7800	1:500	Abcam, Cambridge, UK
TP63	ab735	1:100	Abcam, Cambridge, UK
ESR1α	VP-E613	1:500	Vector Laboratories, Peterborough, UK
CDH1	610182	1:300	BD transduction laboratories, California, USA
TWIST1	ab50887	1:500	Abcam, Cambridge, UK
Ki67	VP-K452	1:20	Vector Laboratories, Peterborough, UK
VIM	SC-7557	1:300	Santa Cruz Biotechnology, Texas, USA
SNAI2	C19G7	1:100	Cell Signalling Technology, Massachusetts, USA
ZEB1	NBP1-05987	1:500	Novus Biologicals, Colorado, USA
ZEB2	NBP1-82991	1:500	Novus Biologicals, Colorado, USA
RAD51	GTX70230	1:100	Genetex Inc., Texas, USA
γH2AX	05-636	1:200	Merck Millipore, Maddachusetts, USA
PAR	4336-BPC-100	1:1000	Trevigen, Maryland, USA
PARP-1	9452	1:1000	Cell Signalling Technology, Massachusetts, USA
VCL	ab18058	1:5000	Abcam, Cambridge, UK
Mouse HRP-conjugated	7074	1:2000	Cell Signalling Technology, Massachusetts, USA
Rabbit HRP-conjugated	7076	1:2000	Cell Signalling Technology, Massachusetts, USA
Mouse Biotinylated	PK-2200	1:200	Vector Laboratories, Peterborough, UK
Rabbit Biotinylated	E0432	1:200	DAKO/Agilent, Santa Clara, USA
Goat Biotinylated	E0466	1:200	DAKO/Agilent, Santa Clara, USA

Taqman probes for qRT-PCR	
Target	Assay ID
Actb	Mm00607939_s1
Abcb1a	Mm00440761_m1
Abcb1b	Mm00440736_m1
Abcg2	Mm00496364_m1
Gapdh	Mm99999915_g1

HRP-conjugated antibodies were used as secondary antibodies in western analysis; biotinylated antibodies were used as secondary antibodies in immunohistochemical analysis. Probes were purchased from Thermo Fisher Scientific. *Actb* and *Gapdh* were used as endogenous controls.

### Immunofluorescence

RAD51 and γH2AX staining used mouse monoclonal antibodies, modifying the protocol described in [40]. Antigen retrieval was performed by microwaving under pressure in Citrate buffer (DAKO). Slides were then incubated with 0.2% Triton for 20 minutes, washed in PBS and treated with DNAse I for 1 hour at 37° C. This was followed by incubation with immunofluorescence buffer (IFF;1% bovine serum albumin, 2% FBS in PBS) for 1 hour at room temperature (RT). The primary antibody diluted in IFF was applied overnight at 4° C, washed with PBS followed by anti-mouse secondary antibody (M.O.M kit, Vector labs, 1:250 dilution) for 30 minutes at RT. Signal was amplified using ABC reagents (Vector labs) for 30 minutes at RT. Sections were then incubated with TSA reagents (PerkinElmer) for 10 minutes at room temperature and washed in DAPI (1:10,000 dilution) for 15 minutes. Sections were fixed with 4% PFA and mounted. Images were analysed by manually counting cells using Image J software.

### RNA expression analysis

For qRT-PCR analysis of gene expression, frozen tumour material was prepared using the Maxwell SimplyRNA LEV Tissue Kit for automated extraction of total RNA (Promega, UK). Briefly, a micro-pestle was used to grind frozen tumour material, on dry ice, prior to adding homogenisation buffer containing 1-Thioglycerol and an equal volume of lysis solution, and the relevant program used for automated RNA extraction with DNase1 treatment. Samples were stored at −80 ° C until used for cDNA synthesis, where 1 µg of RNA per sample was converted to cDNA using the Quantitect cDNA Synthesis Kit (Qiagen, UK). qPCR reactions were performed using the cDNA as described previously [41]. Details of Taqman probes can be found in Table 3. All results were calculated using the ∆−∆_Ct_ method. Data were expressed as the mean fold gene expression difference over comparator samples with 95% confidence intervals.

For gene expression analysis by RNA-seq, tumours from all response groups were lysed in chilled Qiazol in a Qiagen Tissue lyser using stainless steel beads. The lysate was incubated at RT for 5 minutes. One-fifth volume of chloroform was added and the sample was then vortexed for 15 seconds followed by a further 3 minutes incubation at RT. Phase separation was performed by 15 minutes centrifugation at 13,000 RPM at 4° C. The upper aqueous phase was loaded into a Qiacube and RNA was extracted using the Qiagen miRNeasy kit with on-column DNase1 treatment. The quality of RNA was analysed on the Agilent 2100 bioanalyser using Agilent RNA 6000 Nano chip and small RNA chips. 5 µg was submitted for library preparation and sequencing.

### Library preparation for RNA-seq

Samples were riboRNA depleted using Ribo-Zero™ rRNA Removal Kit (Epicentre) according to the manufacturer’s instructions. The final depleted RNA was checked by Qubit assay and Bioanalyzer RNA pico chips and 50 ng used in the ScriptSeq v2 protocol (Epicentre). Libraries were quantified by Qubit assay and checked for size range on a Bioanalyser DNA high sensitivity chip. The libraries were quantified by qPCR, using an Illumina Library Quantification Kit (KAPA, KK4854,) on a Roche LightCycler 480II system.

Small RNA libraries were prepared from total RNA using NEBNext^®^ Multiplex Small RNA Library Prep Set for Illumina^®^ according to the manufacturer’s instructions. Samples were pooled on an equimolar basis and the small RNA fraction selected using Sage Pippin Prep (selecting between 130-170 bp).

### RNA-seq

RNA-seq libraries were sequenced using 100 bp paired-end reads on the Illumina Hi-Seq platform at 150-fold coverage (100 million reads, 3 samples per lane). Sequencing of small RNAs was performed on eight lanes of the Hi-Seq platform using 1× 75bp single reads. Sequencing reads which were of low quality or contained polyA and adapters were pre-filtered before mapping. Filtered reads were mapped to the mm10 mouse genome using Tophat [42] and gene-level counts generated using HTSeq [43]. Differentially-expressed genes were identified using the DESeq R package [44].

### Pharmacokinetics/pharmacodynamics of olaparib in *Brca2/p53* mutant mammary tumours

Samples were analysed for olaparib using a protein precipitation extraction procedure, followed by LC-MS/MS detection. A stock (2 mM) of the analytical standard was prepared using DMSO and subsequently used to produce spiking solutions. 47.5 µl of the required blank matrix was aliquoted into a 96 well plate and the matrix was subsequently spiked with 2.5 µl of each dilution to give a final concentration range of 1 nM–10,000 nM. 50 µl of each sample and standards were quenched with acetonitrile with internal standard, mixed, and spun in a centrifuge at 3000 rpm for 15 minutes. 50µl of the supernatant was then diluted 10-fold with deionised water and the samples analysed by LC-MS/MS using Masslynx and processed using Quanlynx.

For analysis of poly(ADP-ribose) (PAR) and PARP-1 levels flash-frozen pieces of tumor were lysed in ice-cold buffer containing Tris–NaCl pH7.5 20 mmol/L, NaCl 137 mmol/L, NP40 1%, glycerol 10%, supplemented with NaF 50 mmol/L, Na_3_VO_4_ 1 mmol/L, Protease complete Inhibitor tablet (Roche 1836145), Phosphatase inhibitor cocktails 2 and 3 (Sigma, P0044 and P5726). Homogenization was performed 3 times using Fastprep tubes (MP Biomedicals #6910-500) and MP Biomedicals Fast Prep-24 machine. All samples were sonicated for 30 seconds using high amplitude (Diagenode), centrifuged at 13,000rpm 4° C for 10 minutes and the supernatants were collected. Protein concentration was calculated using Pierce Protein Assay Reagent A plus BCA Protein Assay Reagent B. A total of 40 µg of protein were separated on 4–12% SDS-PAGE gels (Invitrogen) at 180V (for 1 hour) in 1× NUPAGE MES SDS running buffer (Invitrogen) in the presence of NuPAGE antioxidant (Invitrogen). Proteins were electrotransferred to 0.2 um nitrocellulose membranes (Invitrogen) using an Iblot dry blotting system (Invitrogen) (20V for 7 min). Membranes were blocked for 1 hour in 5% milk in Tris-buffered saline (TBS)-Tween and then hybridized using the primary antibodies overnight at 4^o^ C in 5% BSA TBS-Tween: rabbit anti-PAR, rabbit anti-PARP1, mouse anti-VCL. Mouse and rabbit horseradish peroxidase (HRP)-conjugated secondary antibodies were diluted in 5% milk in TBS-Tween, incubated with blots for 1 hour and proteins were detected with SuperSignal West Dura Chemiluminescent Substrate reagent (Pierce Thermo Scientific). See Table 3 for antibody details. Biomarker signals were quantified using Genetools software and normalized to VCL control.

### Statistics

Statistical analysis was performed using the non-parametric two-tailed Mann–Whitney *U* test, except for comparison of tumour proportions, which was conducted by Chi Squared test and analysis of PAR and PARP-1 levels, which were calculated using VCL-normalised logged (log_10_) data by ANOVA. *P* ≤ 0.05 was considered to be statistically significant. All error bars on graphs represent standard deviations.

## Abbreviations

AC(NST): Adenocarcinoma/invasive ductal carcinoma of no special type
AME: Adenomyoepithelioma
ASQC: Adenosquamous carcinoma
EMT: Epithelial-mesenchymal transition
HR: Homologous recombination
HRR: Homologous recombination repair
MSCC: Carcinosarcoma/metaplastic spindle cell carcinoma
PAR: Poly (ADP-ribose)
PARP: Poly (ADP-ribose) polymerase
P-gp: Phosphoglycoprotein
PK: Pharmacokinetic
PD: Pharmacodynamic

## References

[1] Bryant HE, Schultz N, Thomas HD, Parker KM, Flower D, Lopez E, Kyle S, Meuth M, Curtin NJ, Helleday T (2005). Specific killing of BRCA2-deficient tumours with inhibitors of poly(ADP-ribose) polymerase. Nature.

[2] Farmer H, McCabe N, Lord CJ, Tutt ANJ, Johnson DA, Richardson TB, Santarosa M, Dillon KJ, Hickson I, Knights C, Martin NMB, Jackson SP, Smith GCM (2005). Targeting the DNA repair defect in BRCA mutant cells as a therapeutic strategy. Nature.

[3] Hay T, Matthews JR, Pietzka L, Lau A, Cranston A, Nygren AOH, Douglas-Jones A, Smith GCM, Martin NMB, O’Connor M, Clarke AR (2009). Poly(ADP-Ribose) Polymerase-1 Inhibitor Treatment Regresses Autochthonous Brca2/p53-Mutant Mammary Tumors *In vivo* and Delays Tumor Relapse in Combination with Carboplatin. Cancer Research.

[4] Rottenberg S, Jaspers JE, Kersbergen A, van der Burg E, Nygren AOH, Zander SAL, Derksen PWB, de Bruin M, Zevenhoven J, Lau A, Boulter R, Cranston A, O’Connor MJ (2008). High sensitivity of BRCA1-deficient mammary tumors to the PARP inhibitor AZD2281 alone and in combination with platinum drugs. Proceedings of the National Academy of Sciences of the United States of America.

[5] McCabe N, Turner NC, Lord CJ, Kluzek K, Bialkowska A, Swift S, Giavara S, O’Connor MJ, Tutt AN, Zdzienicka MZ, Smith GC, Ashworth A (2006). Deficiency in the repair of DNA damage by homologous recombination and sensitivity to poly(ADP-ribose) polymerase inhibition. Cancer Res.

[6] Ledermann J, Harter P, Gourley C, Friedlander M, Vergote I, Rustin G, Scott CL, Meier W, Shapira-Frommer R, Safra T, Matei D, Fielding A, Spencer S (2014). Olaparib maintenance therapy in patients with platinum-sensitive relapsed serous ovarian cancer: a preplanned retrospective analysis of outcomes by BRCA status in a randomised phase 2 trial. Lancet Oncol.

[7] Barber LJ, Sandhu S, Chen L, Campbell J, Kozarewa I, Fenwick K, Assiotis I, Rodrigues DN, Reis Filho JS, Moreno V, Mateo J, Molife LR, De Bono J (2013). Secondary mutations in BRCA2 associated with clinical resistance to a PARP inhibitor. The Journal of Pathology.

[8] Jaspers JE, Sol W, Kersbergen A, Schlicker A, Guyader C, Xu G, Wessels L, Borst P, Jonkers J, Rottenberg S (2015). BRCA2-Deficient Sarcomatoid Mammary Tumors Exhibit Multidrug Resistance. Cancer Research.

[9] Jaspers JE, Kersbergen A, Boon U, Sol W, van Deemter L, Zander SA, Drost R, Wientjens E, Ji J, Aly A, Doroshow JH, Cranston A, Martin NMB (2013). Loss of 53BP1 Causes PARP Inhibitor Resistance in Brca1-Mutated Mouse Mammary Tumors. Cancer Discovery.

[10] Edwards SL, Brough R, Lord CJ, Natrajan R, Vatcheva R, Levine DA, Boyd J, Reis-Filho JS, Ashworth A (2008). Resistance to therapy caused by intragenic deletion in BRCA2. Nature.

[11] Pettitt SJ, Rehman FL, Bajrami I, Brough R, Wallberg F, Kozarewa I, Fenwick K, Assiotis I, Chen L, Campbell J, Lord CJ, Ashworth A (2013). A genetic screen using the PiggyBac transposon in haploid cells identifies Parp1 as a mediator of olaparib toxicity. PLoS One.

[12] Al Sayed AD, El Weshi AN, Tulbah AM, Rahal MM, Ezzat AA (2006). Metaplastic carcinoma of the breast Clinical presentation, treatment results and prognostic factors. Acta Oncologica.

[13] Hennessy BT, Giordano S, Broglio K, Duan Z, Trent J, Buchholz TA, Babiera G, Hortobagyi GN, Valero V (2006). Biphasic metaplastic sarcomatoid carcinoma of the breast. Annals of Oncology.

[14] Molyneux G, Geyer FC, Magnay FA, McCarthy A, Kendrick H, Natrajan R, MacKay A, Grigoriadis A, Tutt A, Ashworth A, Reis-Filho JS, Smalley MJ (2010). BRCA1 Basal-like Breast Cancers Originate from Luminal Epithelial Progenitors and Not from Basal Stem Cells. Cell Stem Cell.

[15] Melchor L, Molyneux G, Mackay A, Magnay F-A, Atienza M, Kendrick H, Nava-Rodrigues D, Angeles Lopez-Garcia M, Milanezi F, Greenow K, Robertson D, Palacios J, Reis-Filho JS (2014). Identification of cellular and genetic drivers of breast cancer heterogeneity in genetically engineered mouse tumour models. The Journal of Pathology.

[16] Oplustilova L, Wolanin K, Mistrik M, Korinkova G, Simkova D, Bouchal J, Lenobel R, Bartkova J, Lau A, O’Connor MJ, Lukas J, Bartek J (2012). Evaluation of candidate biomarkers to predict cancer cell sensitivity or resistance to PARP-1 inhibitor treatment. Cell Cycle.

[17] O’Connor LO, Rulten SL, Cranston AN, Odedra R, Brown H, Jaspers JE, Jones L, Knights C, Evers B, Ting A, Bradbury RH, Pajic M, Rottenberg S (2016). The PARP Inhibitor AZD2461 Provides Insights into the Role of PARP3 Inhibition for Both Synthetic Lethality and Tolerability with Chemotherapy in Preclinical Models. Cancer Research.

[18] Sun CK, Zhang F, Xiang T, Chen Q, Pandita TK, Huang Y, Hu MC, Yang Q (2014). Phosphorylation of ribosomal protein S6 confers PARP inhibitor resistance in BRCA1-deficient cancers. Oncotarget.

[19] Fong PC, Boss DS, Yap TA, Tutt A, Wu P, Mergui-Roelvink M, Mortimer P, Swaisland H, Lau A, O’Connor MJ, Ashworth A, Carmichael J, Kaye SB (2009). Inhibition of Poly(ADP-Ribose) Polymerase in Tumors from BRCA Mutation Carriers. The New England Journal of Medicine.

[20] Tutt A, Robson M, Garber JE, Domchek SM, Audeh MW, Weitzel JN, Friedlander M, Arun B, Loman N, Schmutzler RK, Wardley A, Mitchell G, Earl H (2010). Oral poly(ADP-ribose) polymerase inhibitor olaparib in patients with BRCA1 or BRCA2 mutations and advanced breast cancer: a proof-of-concept trial. Lancet.

[21] Arumugam T, Ramachandran V, Fournier KF, Wang H, Marquis L, Abbruzzese JL, Gallick GE, Logsdon CD, McConkey DJ, Choi W (2009). Epithelial to Mesenchymal Transition Contributes to Drug Resistance in Pancreatic Cancer. Cancer Research.

[22] Wang ZW, Li YW, Kong D, Banerjee S, Ahmad A, Azmi AS, Ali S, Abbruzzese JL, Gallick GE, Sarkar FH (2009). Acquisition of Epithelial-Mesenchymal Transition Phenotype of Gemcitabine-Resistant Pancreatic Cancer Cells Is Linked with Activation of the Notch Signaling Pathway. Cancer Research.

[23] McConkey DJ, Choi W, Marquis L, Martin F, Williams MB, Shah J, Svatek R, Das A, Adam L, Kamat A, Siefker-Radtke A, Dinney C (2009). Role of epithelial-to-mesenchymal transition (EMT) in drug sensitivity and metastasis in bladder cancer. Cancer Metastasis Reviews.

[24] Frederick BA, Helfrich BA, Coldren CD, Zheng D, Chan D, Bunn PA, Raben D (2007). Epithelial to mesenchymal transition predicts gefitinib resistance in cell lines of head and neck squamous cell carcinoma and non-small cell lung carcinoma. Molecular Cancer Therapeutics.

[25] Ahmed N, Abubaker K, Findlay J, Quinn M (2010). Epithelial Mesenchymal Transition and Cancer Stem Cell-Like Phenotypes Facilitate Chemoresistance in Recurrent Ovarian Cancer. Current Cancer Drug Targets.

[26] Latifi A, Abubaker K, Castrechini N, Ward AC, Liongue C, Dobill F, Kumar J, Thompson EW, Quinn MA, Findlay JK, Ahmed N (2011). Cisplatin Treatment of Primary and Metastatic Epithelial Ovarian Carcinomas Generates Residual Cells With Mesenchymal Stem Cell-Like Profile. Journal of Cellular Biochemistry.

[27] McMorrow T, Gaffney MM, Slattery C, Campbell E, Ryan MP (2005). Cyclosporine A induced epithelial-mesenchymal transition in human renal proximal tubular epithelial cells. Nephrology, Dialysis, Transplantation.

[28] Li QQ, Xu JD, Wang WJ, Cao XX, Chen Q, Tang F, Chen ZQ, Liu XP, Xu ZD (2009). Twist1-Mediated Adriamycin-Induced Epithelial-Mesenchymal Transition Relates to Multidrug Resistance and Invasive Potential in Breast Cancer Cells. Clinical Cancer Research.

[29] Schafer ZT, Grassian AR, Song L, Jiang Z, Gerhart-Hines Z, Irie HY, Gao S, Puigserver P, Brugge JS (2009). Antioxidant and oncogene rescue of metabolic defects caused by loss of matrix attachment. Nature.

[30] Cassim S, Raymond VA, Dehbidi-Assadzadeh L, Lapierre P, Bilodeau M (2018). Metabolic reprogramming enables hepatocarcinoma cells to efficiently adapt and survive to a nutrient-restricted microenvironment. Cell Cycle.

[31] Cassim S, Raymond VA, Lacoste B, Lapierre P, Bilodeau M (2018). Metabolite profiling identifies a signature of tumorigenicity in hepatocellular carcinoma. Oncotarget.

[32] Vander Heiden MG, Cantley LC, Thompson CB (2009). Understanding the Warburg effect: the metabolic requirements of cell proliferation. Science.

[33] Gottesman MM (2002). Mechanisms of cancer drug resistance. Annual Review of Medicine.

[34] Allison Stewart C, Tong P, Cardnell RJ, Sen T, Li L, Gay CM, Masrorpour F, Fan Y, Bara RO, Feng Y, Ru Y, Fujimoto J, Kundu ST (2017). Dynamic variations in epithelial-to-mesenchymal transition (EMT), ATM, and SLFN11 govern response to PARP inhibitors and cisplatin in small cell lung cancer. Oncotarget.

[35] Pu H, Horbinski C, Hensley PJ, Matuszak EA, Atkinson T, Kyprianou N (2014). PARP-1 regulates epithelial-mesenchymal transition (EMT) in prostate tumorigenesis. Carcinogenesis.

[36] Selbert S, Bentley DJ, Melton DW, Rannie D, Lourenco P, Watson CJ, Clarke AR (1998). Efficient BLG-Cre mediated gene deletion in the mammary gland. Transgenic Research.

[37] Cheung AMY, Hande MP, Jalali F, Tsao MS, Skinnider B, Hirao A, McPherson JP, Karaskova J, Karaskova J, Suzuki A, Wakeham A, You-Ten A, Elia A (2002). Loss of Brca2 and p53 synergistically promotes genomic instability and deregulation of T-cell apoptosis. Cancer Research.

[38] Jonkers J, Meuwissen R, van der Gulden H, Peterse H, van der Valk M, Berns A (2001). Synergistic tumor suppressor activity of BRCA2 and p53 in a conditional mouse model for breast cancer. Nature Genetics.

[39] Lakhani SR, Ellis IO, Schnitt SJ, Tan PH, van de Vijver MJ (2012). WHO Classification of Tumours of the Breast: International Agency for Research on Cancer.

[40] Graeser M, McCarthy A, Lord CJ, Savage K, Hills M, Salter J, Orr N, Parton M, Smith IE, Reis JS, Dowsett M, Ashworth A, Turner NC (2010). A Marker of Homologous Recombination Predicts Pathologic Complete Response to Neoadjuvant Chemotherapy in Primary Breast Cancer. Clinical Cancer Research.

[41] Kendrick H, Regan JL, Magnay FA, Grigoriadis A, Mitsopoulos C, Zvelebil M, Smalley MJ (2008). Transcriptome analysis of mammary epithelial subpopulations identifies novel determinants of lineage commitment and cell fate. BMC Genomics.

[42] Trapnell C, Pachter L, Salzberg SL (2009). TopHat: discovering splice junctions with RNA-Seq. Bioinformatics.

[43] Anders S, Pyl PT, Huber W (2015). HTSeq--a Python framework to work with high-throughput sequencing data. Bioinformatics.

[44] Anders S, Huber W (2010). Differential expression analysis for sequence count data. Genome Biology.

[45] Sleeman KE, Kendrick H, Ashworth A, Isacke CM, Smalley MJ (2006). CD24 staining of mouse mammary gland cells defines luminal epithelial, myoepithelial/basal and non-epithelial cells. Breast Cancer Res.

[46] Smalley MJ, Titley J, O’Hare MJ (1998). Clonal characterization of mouse mammary luminal epithelial and myoepithelial cells separated by fluorescence-activated cell sorting. *In Vitro* Cell Dev Biol Anim.

[47] Soady KJ, Kendrick H, Gao Q, Tutt A, Zvelebil M, Ordonez LD, Quist J, Tan DW, Isacke CM, Grigoriadis A, Smalley MJ (2015). Mouse mammary stem cells express prognostic markers for triple-negative breast cancer. Breast Cancer Res.

